# Development and internal validation of a prognostic nomogram incorporating F-NLR and HAGR scores in patients with oral squamous cell carcinoma: a single-center retrospective cohort study

**DOI:** 10.3389/fonc.2026.1862842

**Published:** 2026-07-02

**Authors:** Yunqi Chen, Zhiqiang Pan, Luwen Song, Zhenghao Ma, Kai Hu, Mo Chen, Dongkun Yang, Jiancheng Li, Lina Jiang

**Affiliations:** 1 Department of Oral and Maxillofacial Surgery, The First Affiliated Hospital of Bengbu Medical University, Bengbu, Anhui, China; 2 School of Stomatology, Bengbu Medical University, Bengbu, Anhui, China

**Keywords:** F-NLR, HAGR, oral squamous cell carcinoma, predictive model, prognosis

## Abstract

**Background and objective:**

Traditionally, the survival prognosis for patients with oral squamous cell carcinoma (OSCC) has predominantly relied on the TNM staging system, which rarely accounts for the biological heterogeneity of individual patients. This study aimed to construct a novel individualized prognostic model for patients with OSCC by integrating traditional clinical parameters with a systemic inflammatory marker fibrinogen-to-neutrophil-lymphocyte ratio (F-NLR) and a nutritional-metabolic indicator the hemoglobin-albumin-globulin ratio (HAGR).

**Methods:**

We retrospectively analyzed the clinical data of 292 patients with OSCC who underwent radical surgical resection at a single center. The optimal cut-off values for continuous variables, including F-NLR and HAGR, were determined using the Youden index derived from receiver operating characteristic (ROC) curves. Univariate and multivariate Cox proportional hazards regression analyses were performed to identify independent prognostic factors, which were subsequently used to construct a nomogram predicting 1-, 3-, and 5-year cancer-specific survival (CSS) rates. The model underwent internal validation only; external validation was not performed.

**Results:**

During a median follow-up of 41 months, 119 cancer-related deaths were observed. Multivariate analysis identified age, history of precancerous lesions, N classification, postoperative adjuvant therapy, and F-NLR and HAGR scores as independent prognostic factors for OSCC. The nomogram demonstrated a C-index of 0.73, with areas under the curve (AUC) for predicting 1-, 3-, and 5-year CSS of 0.798, 0.754, and 0.836, respectively, indicating acceptable model discrimination. Calibration plots revealed high consistency between the nomogram-predicted probabilities and actual survival observations. Furthermore, DCA suggested a potential net benefit when utilizing this nomogram to guide clinical interventions across a broad range of threshold probabilities.

**Conclusion:**

The proposed nomogram, incorporating F-NLR and HAGR scores alongside traditional clinical parameters, demonstrates acceptable predictive accuracy and promising potential for individualized risk stratification in patients with OSCC. However, further external validation in multi-center cohorts is required before it can be routinely recommended for clinical decision-making.

## 1 Introduction

As the most prevalent malignancy of the head and neck, oral squamous cell carcinoma (OSCC) is characterized by aggressive local invasion and a high propensity for cervical lymph node metastasis (1). Despite substantial recent advancements in multimodal therapies—encompassing surgical resection, radiotherapy, chemotherapy, and immunotherapy—the long-term survival rates for patients with OSCC remain suboptimal (2). In current clinical practice, prognostic evaluation and treatment planning primarily rely on the American Joint Committee on Cancer (AJCC) TNM staging system. However, this system possesses inherent limitations; it relies predominantly on anatomical parameters—such as tumor size, depth of invasion, and lymph node involvement—without accounting for the biological heterogeneity of the host (3). Consequently, anatomical staging alone is insufficient to comprehensively capture the tumor’s true invasive potential (4).

To address these limitations, prognostic nomograms have recently gained prominence in the management of OSCC and head and neck cancers (HNC). By integrating multiple demographic, clinical, and biological variables into a visual and quantitative tool, nomograms offer superior individualized risk stratification compared to traditional staging (5–7). While previous models have successfully incorporated single blood-based markers or general clinical characteristics, many existing nomograms fail to capture the complex, dual-faceted nature of host systemic exhaustion—specifically the interplay between tumor-induced hyperinflammation and severe nutritional depletion.

Tumorigenesis and metastasis are dictated not merely by intrinsic genetic alterations, but are profoundly modulated by this systemic exhaustion (8). Although established inflammatory indices such as the neutrophil-to-lymphocyte ratio (NLR), platelet-to-lymphocyte ratio (PLR), and systemic immune-inflammation index (SII) effectively reflect systemic inflammation, they generally overlook the pro-coagulant state frequently observed in cancer progression. The fibrinogen-to-neutrophil-lymphocyte ratio (F-NLR) overcomes this limitation by simultaneously capturing both inflammatory and coagulation pathways, thus providing a more comprehensive reflection of the “pro-tumorigenic” and “immunosuppressive” microenvironment (9). Similarly, while the prognostic nutritional index (PNI), albumin-to-globulin ratio (AGR), and CONUT score are widely used nutritional tools, the hemoglobin-albumin-globulin ratio (HAGR) may offer specific prognostic insights in OSCC. By incorporating hemoglobin, HAGR concurrently assesses baseline nutritional reserves, host immune status, and tissue oxygenation capacity (10). This is particularly relevant in OSCC, where feeding difficulties caused by the primary lesion and microenvironmental hypoxia strictly drive tumor aggressiveness.

Beyond systemic indices, the prognosis of OSCC is uniquely influenced by the local mucosal microenvironment. The presence of concurrent precancerous lesions at diagnosis typically indicates that widespread areas of the oral mucosa have acquired malignant potential, aligning with the concept of “field cancerization” (11).

The incremental value of our proposed nomogram lies in bridging the gap between systemic biological profiling and oral-specific clinical realities. Unlike previously published models that rely solely on universal blood markers or TNM stages (12), our model synthesizes high-fidelity systemic indicators (F-NLR and HAGR) with highly specific local clinicopathological features (history of precancerous lesions). To the best of our knowledge, based on current literature, the concurrent integration of these dual systemic markers and specialized local risk factors into a cohesive prognostic evaluation system for OSCC remains largely underexplored (9).

Therefore, this retrospective study was conducted with specific objectives. The primary objective was to investigate the independent prognostic value of F-NLR and HAGR in patients with OSCC following radical surgical resection. The secondary objective was to develop and internally validate a novel, simple, and non-invasive prognostic nomogram integrating these hematological markers with local clinical features, aiming to provide a reliable tool for individualized survival prediction and tailored clinical decision-making.

## 2 Materials and methods

### 2.1 Study design and participants

This was a retrospective cohort study conducted in accordance with the Transparent Reporting of a multivariable prediction model for Individual Prognosis or Diagnosis (TRIPOD) guidelines. We retrospectively reviewed the clinical data of patients with OSCC who underwent inpatient radical surgical resection at the First Affiliated Hospital of Bengbu Medical University between January 2020 and December 2022.

The inclusion criteria were as follows: (1) histopathologically confirmed primary OSCC, restricted exclusively to squamous cell carcinoma (OSCC); (2) surgical resection as the primary therapeutic modality; (3) availability of complete and reliable treatment and follow-up data; and (4) age greater than 18 years. Patients were excluded if they met any of the following criteria: (1) received neoadjuvant chemoradiotherapy prior to surgery; (2) death from non-cancer-related causes within 30 days postoperatively (perioperative mortality); (3) presence of adverse clinical features, including suspected distant metastasis on preoperative imaging, incomplete resection, or positive surgical margins; and (4) a prior history of other malignancies. During the designated study period, a total of 292 consecutive patients with OSCC who strictly met all predefined inclusion criteria and possessed complete baseline clinicopathological and hematological data were identified and enrolled in this retrospective cohort. Patients with incomplete medical records, those lost to immediate postoperative follow-up, or those meeting any of the exclusion criteria were not extracted for the final complete-case analysis. No imputation methods were utilized for missing data.

### 2.2 Clinical characteristics and blood sampling

Clinical parameters for all patients were comprehensively recorded, encompassing sex, age, tumor site, history of precancerous lesions (e.g., leukoplakia, erythroplakia, lichen planus), betel quid chewing history, deleterious habits (tobacco and alcohol consumption), systemic chronic comorbidities (hypertension, diabetes mellitus, cardiovascular disease, cerebral infarction), T and N classifications, as well as the history of postoperative adjuvant radiotherapy, chemotherapy, and immunotherapy.

Routine preoperative blood samples were collected strictly within 7 days prior to surgical intervention. If multiple blood tests were performed, the results closest to the date of surgery were utilized to accurately reflect the immediate baseline systemic state. Hematological indices were derived from routine complete blood counts (CBC), comprehensive metabolic panels, and coagulation profiles. The neutrophil-to-lymphocyte ratio (NLR) was calculated using absolute neutrophil counts divided by absolute lymphocyte counts. The hemoglobin-albumin-globulin ratio (HAGR) was calculated as: [hemoglobin (g/L) × serum albumin (g/L)]/serum globulin (g/L).

### 2.3 Follow-up and endpoints

The primary endpoint of this study was cancer-specific survival (CSS), defined as the interval from the date of surgical resection to the date of death specifically attributable to OSCC. No patients were completely lost to follow-up during the study period, yielding a follow-up completeness rate of 100%. Patients who were alive without disease progression at the end of the study were censored at the date of their last clinical contact. While we acknowledge that censoring non-cancer deaths may theoretically introduce competing risk bias, this approach is standard for calculating CSS and appropriately isolating the biological impact of OSCC.

The final follow-up data cutoff date was December 31, 2025. The median follow-up time was 41 months (range: [2.0 - 70.0] months). A total of 173 patients were censored, yielding a follow-up completeness rate of 100%.

### 2.4 Grouping criteria and variable categorization

To facilitate practical clinical application and scoring within the nomogram, continuous variables (NLR, fibrinogen [FIB], and HAGR) were categorized. While this categorization facilitates intuitive clinical scoring, we acknowledge that it inherently entails a potential loss of continuous statistical information and cutoff instability. The optimal cutoff values were determined using the maximum Youden index derived from time-dependent receiver operating characteristic (ROC) curves specifically for 3-year CSS. The time-dependent ROC curve analysis for determining the optimal cutoff values of continuous variables was performed using the ‘timeROC’ package in R software.

Based on this method, the optimal cutoff for FIB was 3.74 g/L, and for NLR, it was 1.6. The F-NLR scoring system was constructed reproducibly as follows: a score of 2 was assigned if both FIB ≥ 3.74 g/L and NLR ≥ 1.6; a score of 1 if only one indicator was elevated; and a score of 0 if both were below their thresholds. Similarly, the optimal threshold for HAGR was determined to be 306.2. This statistical cutoff aligns well with clinical states of cachexia and hypoalbuminemia. Accordingly, patients were dichotomized: a score of 1 (high-risk) was assigned to the low-HAGR group (≤ 306.2), denoting nutritional depletion, while a score of 0 (low-risk) was assigned to the high-HAGR group (> 306.2).

### 2.5 Statistical analysis

Statistical analyses were performed using SPSS software (version 27.0) and R software (version 4.5.1). Univariate survival analysis (Kaplan-Meier estimates and log-rank tests) and multivariable Cox proportional hazards regression were conducted using SPSS. Variables demonstrating a P-value < 0.05 in the univariate analysis were subsequently entered into the multivariable model. Predictors that maintained statistical significance (P < 0.05) in the multivariable analysis were identified as independent prognostic factors. Subsequently, R software was utilized to construct the final nomogram based on these independent factors. The proportional hazards (PH) assumption was rigorously tested for all variables in the final Cox model using Schoenfeld residuals via R software; no significant violations were observed (all P > 0.05). Notably, with 119 cancer-related events and 6 variables incorporated into the final model, our study achieved an Events Per Variable (EPV) ratio of 19.8. This safely exceeds the recommended rule-of-thumb of 10 EPV, substantially minimizing the risk of overfitting.

A prognostic nomogram for 1-, 3-, and 5-year CSS was constructed using the ‘rms’ package. Model discrimination was assessed using Harrell’s concordance index (C-index) and time-dependent ROC curves (via the ‘timeROC’ package), with 95% confidence intervals (CIs) calculated for the area under the curve (AUC). Both the C-index and calibration curves were optimism-corrected using internal validation via 1,000 bootstrap resamples to ensure robustness. The model underwent internal validation only; external validation was not performed.

For risk stratification, the comprehensive risk score (linear predictor) for each patient was calculated based on the final Cox model. Patients were stratified into high-risk and low-risk cohorts using the median risk score as the cutoff value. Kaplan-Meier survival curves were plotted, accompanied by a number-at-risk table, and group differences were compared using the log-rank test. Finally, Decision Curve Analysis (DCA) was performed using the ‘ggDCA’ package to evaluate the clinical net benefit. All tests were two-sided, and P < 0.05 was considered statistically significant.

## 3 Results

### 3.1 Baseline characteristics of the study population

During a median follow-up of 41 months, the study population consisted of 292 patients with OSCC, comprising 186 males (63.7%) and 106 females (36.3%). The median age was 66 years. The most common tumor subsite was the tongue (43.5%, n=127), followed by the floor of the mouth (17.1%, n=50) and gums (16.4%, n=48). Regarding anatomical staging, the cohort was predominantly early-stage, with 87.3% (n=255) classified as T1/T2, and 67.8% (n=198) presenting without regional lymph node metastasis (N0).

A total of 185 patients (63.4%) received postoperative adjuvant therapy. Due to the retrospective nature of this study, sample size limitations across diverse treatment subsets, and standard multimodal protocols for high-risk patients, adjuvant treatments (radiotherapy, chemotherapy, and immunotherapy) were combined into a single binary variable. However, we acknowledge that interpreting this combined variable must be done cautiously, as it is highly prone to confounding by indication. Detailed pathological features (e.g., depth of invasion, perineural invasion, and extranodal extension) were excluded from the primary analysis due to a substantial rate of missing data in this historical cohort. By the end of the follow-up period, 119 cancer-specific deaths were recorded (**Table 1**).

**Table 1:** Baseline clinicopathological characteristics of the study cohort.

Variable	Total cohort (N = 292), n (%)
Age (years) Median (IQR)	66 (53-73)
≤ 59	118 (40.4%)
60-74	110 (37.7%)
≥ 75	64 (21.9%)
Sex	
Female	106 (36.3%)
Male	186 (63.7%)
Site of onset	
Tongue	127 (43.5%)
Floor of mouth	50 (17.1%)
Gums	48 (16.4%)
Palate	31 (10.6%)
Buccal	27 (9.2%)
Posterior molar	9 (3.1%)
T classification	
T1/T2	255 (87.3%)
T3/T4	37 (12.7%)
N classification	
N0	198 (67.8%)
N1-N3	94 (32.2%)
History of precancerous lesions	
No	181 (62.0%)
Yes	111 (38.0%)
Betel quid chewing history	
No	198 (67.8%)
Yes	94 (32.2%)
Postoperative adjuvant therapy	
No	107 (36.6%)
Yes	185 (63.4%)
F-NLR score	
0	85 (29.1%)
1	168 (57.5%)
2	39 (13.4%)
HAGR Median (IQR)	243.41 (199.88 - 298.80)
> 306.2 (Low risk)	70 (24.0%)
≤ 306.2 (High risk)	222 (76.0%)
Continuous Blood Markers	
FIB (g/L), Median (IQR)	2.92 (2.41 - 3.42)
NLR, Median (IQR)	1.90 (1.50 - 2.60)

IQR, interquartile range; F-NLR, fibrinogen-to-neutrophil-lymphocyte ratio; HAGR, hemoglobin-albumin-globulin ratio; FIB, fibrinogen; NLR, neutrophil-to-lymphocyte ratio.

### 3.2 Identification of independent prognostic factors

Univariate log-rank analysis revealed that sex, age, history of precancerous lesions, betel quid chewing history, N classification, postoperative adjuvant therapy, F-NLR, and HAGR scores were significantly associated with cancer-specific survival (CSS) (all P < 0.05) (**Table 2**). Notably, T classification was not statistically significant (P = 0.121). This lack of significance, despite being a central component of OSCC staging, is primarily attributed to the highly skewed distribution of our cohort, where 87.3% of patients were concentrated in the T1/T2 stages, resulting in insufficient statistical power to detect survival differences based solely on tumor size.

**Table 2:** Univariate and multivariable Cox regression analyses for cancer-specific survival in patients with OSCC.

Variable	Univariate analysis		Multivariable analysis	
	HR (95% CI)	P value	HR (95% CI)	P value
Age (years)		<0.001		0.011
≤ 59	1.00 (Reference)		1.00 (Reference)	
60-74	1.329 (0.858-2.057)		1.267 (0.787-2.038)	0.330
≥ 75	2.485 (1.578-3.912)		2.122 (1.270-3.546)	0.004
Sex		0.038		0.866
Female	1.00 (Reference)		1.00 (Reference)	
Male	1.567 (1.025-2.395)		1.040 (0.662-1.634)	
Site of onset		0.670		
Tongue	1.00 (Reference)		-	-
Other sites	1.082 (0.752-1.558)		-	-
T classification		0.121		
T1/T2	1.00 (Reference)		-	-
T3/T4	1.479 (0.896-2.443)		-	-
N classification		0.009		0.042
N0	1.00 (Reference)		1.00 (Reference)	
N1-N3	1.960 (1.185-3.243)		1.760 (1.021-3.034)	
History of precancerous lesions		<0.001		0.010
No	1.00 (Reference)		1.00 (Reference)	
Yes	1.874 (1.305-2.690)		1.717 (1.136-2.595)	
Betel quid chewing history		0.025		0.102
No	1.00 (Reference)		1.00 (Reference)	
Yes	1.524 (1.055-2.202)		1.379 (0.938-2.025)	
Postoperative adjuvant therapy		<0.001		<0.001
Yes	1.00 (Reference)		1.00 (Reference)	
No	2.195 (1.529-3.149)		2.051 (1.386-3.036)	
F-NLR score		<0.001		0.018
0	1.00 (Reference)		1.00 (Reference)	
1	1.501 (0.948-2.376)		1.403 (0.881-2.233)	
2	3.154 (1.829-5.441)		2.240 (1.277-3.929)	
HAGR		<0.001		<0.001
> 306.2	1.00 (Reference)		1.00 (Reference)	
≤ 306.2	2.359 (1.622-3.431)		2.246 (1.512-3.337)	

HR, hazard ratio; CI, confidence interval; CSS, cancer-specific survival. F-NLR, fibrinogen-to-neutrophil-lymphocyte ratio; HAGR, hemoglobin-albumin-globulin ratio; FIB, fibrinogen; NLR, neutrophil-to-lymphocyte ratio. Multivariable model included variables with P < 0.05 in the univariate analysis. “-” indicates that the variable was not included in the multivariable model.

To identify independent prognostic factors, all variables demonstrating a P-value < 0.05 in the univariate analysis—including sex and betel quid chewing history—were entered into the multivariable Cox proportional hazards model. The multivariable analysis confirmed that age, history of precancerous lesions, N classification, postoperative adjuvant therapy, F-NLR score, and HAGR score were independent predictors of CSS (**Table 2**).

### 3.3 Development and validation of the prognostic nomogram

**Figure 1:**
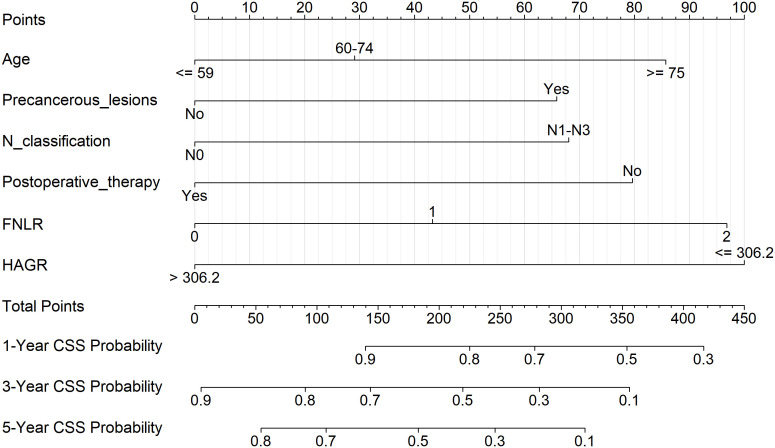
Prognostic nomogram model for patients with OSCC following surgery.

**Figure 2:**
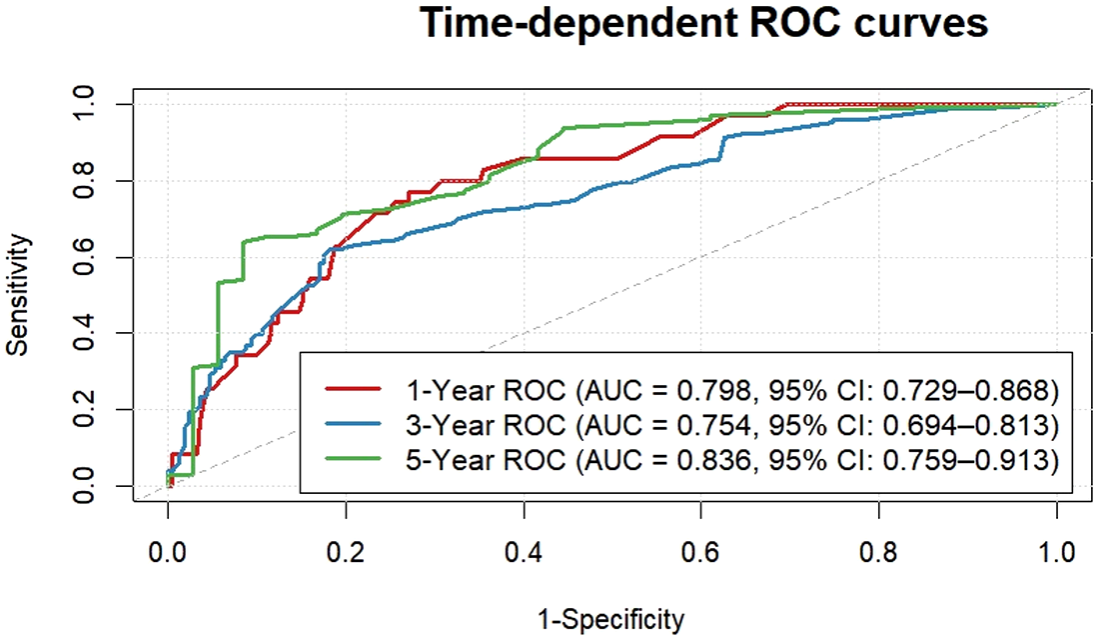
Time-dependent ROC curves evaluating the discriminatory power of the nomogram.

**Figure 3:**
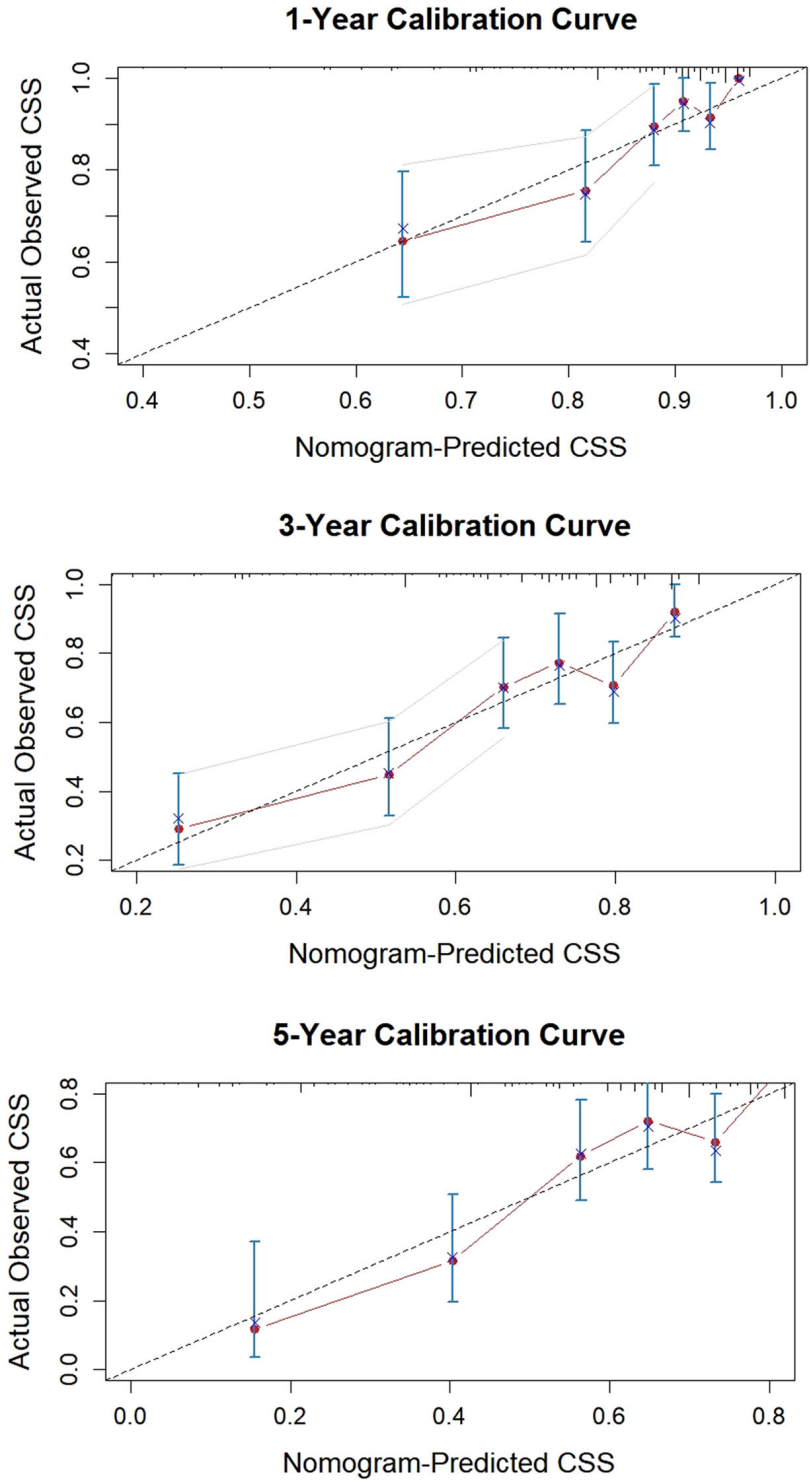
Calibration plots for predicting 1,3,5-year cancer-specific survival.

Based on the independent prognostic factors identified, a nomogram was developed to predict 1-, 3-, and 5-year CSS probabilities (**Figure 1**). The model demonstrated an internally validated C-index of 0.73. Time-dependent ROC curves (**Figure 2**) yielded AUC values of 0.798 (95% CI: 0.729–0.868), 0.754 (95% CI: 0.694–0.813), and 0.836 (95% CI: 0.759–0.913) for 1-, 3-, and 5-year CSS, respectively, indicating acceptable discriminatory power. Furthermore, calibration plots generated for 1-year, 3-year, and 5-year CSS (**Figure 3**) demonstrated excellent agreement between the nomogram-predicted probabilities and the actual survival outcomes observed via the Kaplan-Meier method.

### 3.4 Risk stratification via the nomogram

**Figure 4:**
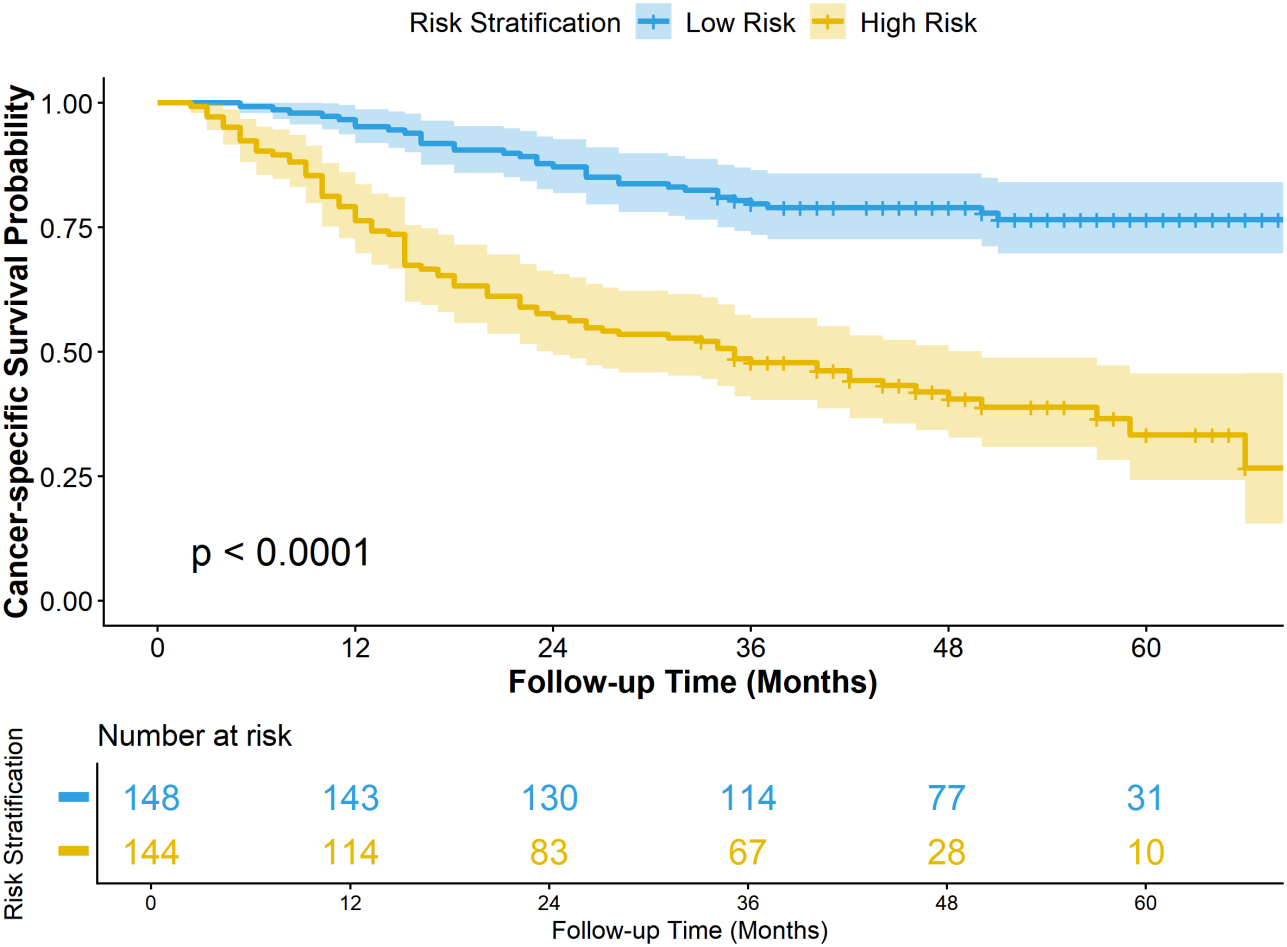
Kaplan-Meier survival analysis for high-risk and low-risk cohorts.

For clinical risk stratification, patients were divided into high-risk and low-risk cohorts using the median linear predictor score as the exact threshold. The high-risk group (n = 144) experienced significantly inferior CSS compared to the low-risk group (n = 148) (Log-rank P < 0.0001) (**Figure 4**). However, because the same dataset was used for both model development and risk stratification, this Kaplan-Meier separation may be optimistic, and we emphasize that external validation is required.

### 3.5 Assessment of clinical utility via decision curve analysis

**Figure 5:**
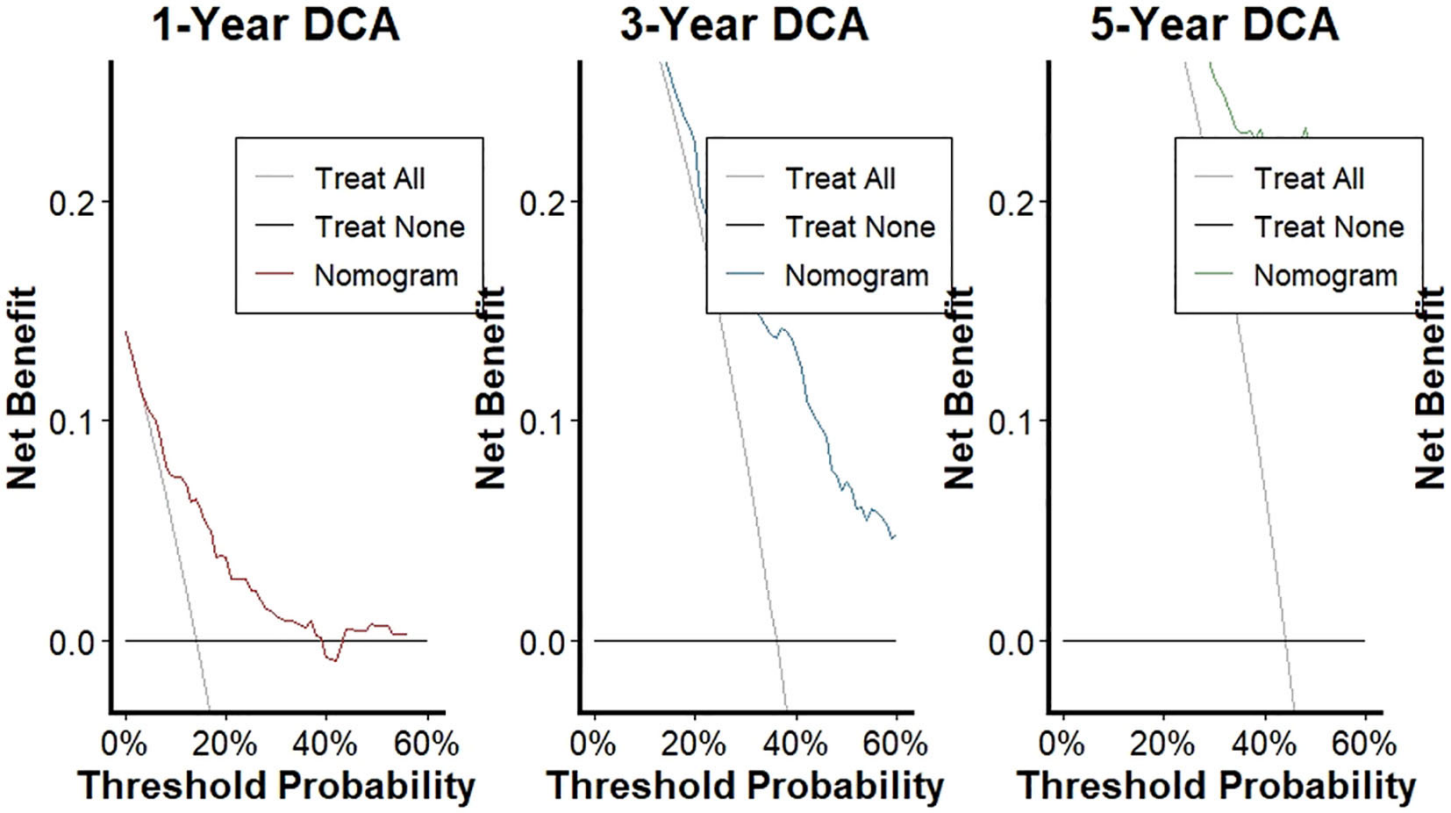
Decision curve analysis (DCA) for predicting 1-, 3-, and 5-year cancer-specific survival.

Decision curve analysis (DCA) was employed to evaluate the clinical net benefit (**Figure 5**). The threshold probability range extending up to 60% indicates that the nomogram is clinically beneficial for both surgeons and patients who are willing to accept a varying risk of cancer-specific mortality before deciding to implement further clinical evaluations or risk-stratified protocols. Within this range, the nomogram-guided strategy offered a higher net benefit than the default “treat-all” or “treat-none” strategies across 1-, 3-, and 5-year predictions. While these results suggest promising clinical utility, they must be interpreted cautiously, as DCA confirming clinical usefulness inherently requires external validation to mitigate the limitations of internal validation.

## 4 Discussion

Accurate prognosis of oral squamous cell carcinoma (OSCC) is core to formulating individualized treatment strategies (13). While traditional TNM staging relies heavily on anatomical measurements (14), individual patient survival is significantly influenced by systemic factors, including age, nutritional status, and systemic inflammation (15). To our knowledge, this study is the first to combine systemic inflammatory (F-NLR) and nutritional (HAGR) markers with highly specific oral risk factors (betel quid chewing and precancerous lesions) into a novel prognostic nomogram. When contextualized within the existing literature, where published prognostic nomograms for OSCC and head and neck cancers typically report C-indices ranging from 0.65 to 0.75, our model achieved a realistic C-index of 0.73. This demonstrates a moderate, acceptable predictive performance, suggesting that the inclusion of F-NLR and HAGR provides important incremental predictive value beyond standard clinicopathological variables alone.

Compared to localized pathological biopsies, elevated F-NLR intuitively reflects a host’s macroscopic “high-inflammation, high-coagulation, and low-immunity” pathophysiological state. While our study was strictly observational and designed to identify clinical associations rather than prove biological mechanisms, existing literature offers compelling mechanistic explanations for these findings. In the complex and highly vascularized maxillofacial region, large aggregations of inflammatory cells at the tumor invasive front are proposed to act as an “accelerant,” degrading the basement membrane and activating osteoclasts, thereby facilitating tumor infiltration into deep muscular spaces and cervical lymph nodes (16) (17). Concurrently, elevated fibrinogen is thought to form a “physical shield” around circulating tumor cells, while lymphocyte depletion reflects the exhaustion of the host’s specific anti-tumor immunity. Thus, F-NLR provides clinicians with a systemic early warning of aggressive, metastasis-prone tumor behavior.

Systemic inflammation is frequently accompanied by severe nutritional depletion, a process quantified by the HAGR score. OSCC primary lesions often cause severe pain, trismus, or dysphagia, leading to significant preoperative malnutrition. In maxillofacial surgery, hypoalbuminemia not only indicates exhausted nutritional reserves but also drastically increases the risk of vascular crises in free-flap reconstructions, salivary fistulas, and delayed wound healing (18). Furthermore, anemia-induced microenvironmental hypoxia is particularly lethal in the high-oxygen-demand head and neck region, potentially driving tumor cells toward a more invasive phenotype. Incorporating HAGR into the prognostic framework objectively reflects this systemic exhaustion.

Regarding anatomical staging, our multivariable model confirmed N classification, rather than T classification, as a profound independent prognosticator. This is highly consistent with the biological reality of OSCC. In our cohort, which predominantly consisted of early-stage (T1/T2) primary tumors, the occurrence of cervical lymph node metastasis (N+) signifies a critical biological shift. It represents not merely an anatomical breach, but a highly aggressive metastatic phenotype that overrides the prognostic impact of local tumor size. The dissemination of tumor cells into the regional lymphatic network actively triggers a systemic immune-tumor interaction (19), perfectly mirroring the hyperinflammatory and immunosuppressive state captured by an elevated F-NLR score.

The detrimental impact of these systemic vulnerabilities is severely amplified when combined with a hostile local mucosal environment. The presence of concurrent precancerous lesions strongly aligns with the “field cancerization” theory, indicating that the entire oral mucosal barrier possesses multicentric malignant potential (20). For these patients, achieving pathologically negative surgical margins at the primary site is clinically insufficient to guarantee long-term survival. The dual burden of an aggressive biological phenotype and a highly unstable local mucosal environment translates to a significantly elevated risk of loco-regional recurrence and the development of second primary malignancies (21).

Furthermore, advanced age emerged as a critical independent risk factor. In real-world maxillofacial surgery, advanced age extends beyond general immune senescence; it is frequently accompanied by systemic microvascular impairments (e.g., atherosclerosis) and profound involution of oral structures, such as severe periodontitis and extensive tooth loss. This baseline impairment often leaves elderly patients in a state of chronic, covert malnutrition even before the tumor develops. Consequently, their physiological tolerance for prolonged procedures—such as extensive tumor resection combined with vascularized free flap reconstructions (e.g., fibula flaps)—is drastically reduced. When confronted with the severe inflammatory burden and nutritional depletion indicated by elevated F-NLR and HAGR, the compensatory capacity of elderly patients is rapidly exhausted.

In the comprehensive management of OSCC, postoperative adjuvant therapy plays a crucial role in eradicating residual microscopic disease. In our cohort, multivariable analysis demonstrated that not receiving adjuvant therapy was an independent risk factor for mortality (HR = 2.051, P < 0.001), indicating that adjuvant therapy provided a significant protective effect and substantially improved cancer-specific survival. However, we acknowledge that interpreting the exact magnitude of this survival benefit must be done cautiously. Due to the retrospective nature of our study, treatment allocation was not randomized and is inherently subject to “confounding by indication.” In clinical practice, adjuvant therapies are typically administered selectively to patients with more advanced, unmeasured disease burdens or adverse baseline biological features (22). The fact that these high-risk patients still exhibited significantly improved survival in our cohort strongly underscores the vital therapeutic efficacy of multimodal treatments. Nevertheless, our nomogram serves primarily as a baseline risk-stratification tool rather than a direct guide for adjuvant treatment allocation, which requires further validation in prospective randomized controlled trials.

By integrating F-NLR and HAGR, our model provides a cost-effective, non-invasive, and convenient alternative to expensive novel biomarkers like next-generation sequencing (NGS), requiring only routine preoperative blood tests (23). To explore its potential practical value, Decision Curve Analysis (DCA) suggested that a nomogram-assisted risk assessment strategy might yield a potential net benefit compared to “treat-all” or “treat-none” approaches across threshold probabilities of 0.1 to 0.7. However, we must strictly avoid overstating its clinical applicability. As a retrospectively derived tool, the model may support individualized risk stratification, but it cannot currently guide treatment allocation—such as determining the extent of surgical margins, the necessity of comprehensive neck dissection, or the administration of adjuvant therapy—without future prospective and external validation (24).

Several significant limitations of this study must be acknowledged. First, the retrospective, single-center design inherently carries risks of selection bias and potential overfitting, particularly exacerbated by the cutoff-based categorization of continuous variables (F-NLR and HAGR). Second, the generalizability of the model is restricted, as our cohort excluded patients receiving neoadjuvant or non-surgical primary therapies, as well as those with recurrent disease, distant metastasis, incomplete resections, and positive margins. Third, relying on a single preoperative blood measurement fails to capture dynamic longitudinal physiological changes during the perioperative and adjuvant therapy periods. Fourth, our analysis lacks several critical pathological variables recognized as major prognostic determinants in OSCC—including depth of invasion, precise margin distance, extranodal extension, perineural invasion, lymphovascular invasion, and tumor grade—due to a substantial rate of missing historical data. Finally, the absence of external validation remains a major limitation.

## 5 Conclusion

The proposed nomogram, which integrates systemic inflammatory and nutritional markers with oral-specific risk factors, showed promising internally validated performance for survival prediction in surgically treated OSCC patients. However, external validation in independent multicenter cohorts is strictly required before the model can be recommended for routine clinical use or therapeutic decision-making.

## Data Availability

The datasets generated and analyzed during the current study are not publicly available in online repositories to strictly protect patient privacy and clinical confidentiality. However, anonymized data can be made available from the corresponding author upon reasonable request and subject to further institutional ethical approval.
